# Chronic anticoagulation therapy associated with increased complications following hemiarthroplasty in hip fracture patients

**DOI:** 10.1007/s00590-025-04578-w

**Published:** 2025-11-04

**Authors:** Soroush Shabani, Julian Wier, Ashley Mulakaluri, David McCavitt, Andrew Duong, Reza Firoozabadi, Joseph Patterson

**Affiliations:** 1 University of Southern California, Los Angeles, USA; 2 University of Washington, Seattle, USA

**Keywords:** Hemiarthroplasty, Anticoagulation, Complications, Infection, Geriatric

## Abstract

**Purpose:**

Femoral neck fractures in older adults are commonly treated with hip hemiarthroplasty (HA). Many older adults who sustain femoral neck fractures are also receiving long-term anticoagulation therapy, which may negatively affect patient outcomes after HA. We sought to investigate whether long-term anticoagulation therapy is associated with increased risks of surgical wound complications among older adults treated with HA for femoral neck fracture.

**Methods:**

Patients ≥ 60 years old who underwent HA within two days of admission for femoral neck fracture between October 1, 2015-December 31, 2021 were identified using codes in the Premier Healthcare Database. Long-term anticoagulation therapy was defined as an active prescription of anticoagulant medication at time of admission. A propensity score for the probability of long-term anticoagulant therapy was used to match patients. The adjusted odds of 90-day infectious surgical wound complications (deep and superficial) and non-infectious surgical wound complications (wound dehiscence, seroma, or hematoma) were determined from the matched patients.

**Results:**

7218 patients on long-term anticoagulation therapy were matched to 7218 patients with no history of anticoagulation therapy. Patients with a history of long-term anticoagulation therapy experienced higher unadjusted rates of non-infectious surgical wound complications (2.15% vs. 1.29%, *p* < 0.001). No difference in the incidence of surgical site infection was observed between groups (1.73% vs. 1.30%, *p* = 0.111). After propensity score matching, patients on long-term anticoagulation therapy had higher odds of non-infectious (adjusted odds ratio [aOR]: 1.65, 95% confidence interval [CI] 1.27–2.15, *p* < 0.001) and infectious (aOR: 1.38, CI 1.05–1.81, *p* = 0.021) surgical wound complications.

**Conclusion:**

Long-term anticoagulation therapy confers an independent risk of wound complications among older adults undergoing HA for femoral neck fracture.

**Supplementary Information:**

The online version contains supplementary material available at 10.1007/s00590-025-04578-w.

## Introduction

Hip fractures represent a global health concern considering their association with high morbidity and mortality [1]. The incidence of hip fractures in the United States (US) is expected to increase from 260,000 to 300,000 admissions annually to 500,000 per year in the US by 2040 as our population ages and remains more active later in life [2–4]. Half of hip fractures involve the femoral neck and the majority of these fractures are displaced. Hemiarthroplasty (HA) is the standard of care for displaced femoral neck fractures in patients with frailty, limited self-sufficiency, impaired mobility, and limited expectancy as hemiarthroplasty is associated with shorter operative time, less blood loss, and fewer complications versus total hip arthroplasty [5–7].

About one in six adults over the age of 65 regularly take anticoagulant medication for conditions that increase their risk of thromboembolic events, including approximately one in three patients with a hip fracture [8, 9]. These medications include vitamin K antagonists, heparin derivatives, low molecular weight heparin, and direct oral anticoagulants [10–12]. The prevalence of comorbid medical conditions associated with increased thromboembolic risk increases gradually with age [10]. As the population ages, the number of older adult patients on chronic anticoagulation therapy who present with a hip fracture is likely to rise [10]. While cessation of these therapies prior to intervention may be considered, the discontinuation of anticoagulation therapy prior to operative fixation increases postoperative cardiovascular or cerebrovascular complications [13].

Currently, there is a lack of robust evidence regarding the effect of preinjury anticoagulation therapy on outcomes in patients with femoral neck fractures undergoing HA. The aim of this study is to investigate whether preinjury anticoagulation therapy is associated with increased risk of infectious and non-infectious surgical site complications among older adults treated with HA for femoral neck fracture.

## Methods

### Data source and study population

Patients who received HA for femoral neck fracture from October 1st, 2015 to December 31, 2021 were identified using the Premier Healthcare Database (PHD). The index HA surgery was identified using International Classification of Diseases, Tenth Revision (ICD-10) Procedural (0SRR0, 0SRS0), and Current Procedural Terminology (27125, 27236) codes. Patients with an admission diagnosis of femoral neck fracture were identified via ICD-10-Clinical Modification (CM) codes (S72.01-4). Patients with a current history of long-term anticoagulation use were identified via ICD-10-CM code (Z79.01). All patients < 60 years old and patients undergoing HA after two days from admission were excluded from final analysis to identify an elderly adult cohort operated on within a clinically appropriate timeframe.

Institutional review board review was not required as all patient information was de-identified in accordance with the Health Insurance Portability and Accountability Act.

### Study endpoints

The primary endpoints were the odds of 90-day infectious (deep and superficial surgical site infection) and non-infectious (wound dehiscence, seroma, or hematoma) surgical site complications. Secondary endpoints were the odds of 90-day irrigation and debridement for primary endpoints, intraoperative transfusion, inpatient medical complications (acute kidney injury, acute respiratory failure, atrial fibrillation with rapid ventricular response, urinary tract infection, pneumonia, and sepsis), 90-day thrombotic complications (myocardial infarction, cerebrovascular accident, or venous thromboembolism [VTE]), inpatient mortality, protracted length of stay (> 50th –percentile, > 5 days), discharge to a skilled nursing facility (SNF), and 90-day readmission. All diagnostic endpoints were identified via ICD-10-CM codes, while procedural endpoints were identified via CPT codes (Supplemental Table 1).

### Propensity score matching

To minimize confounding, a propensity score on the probability of preinjury chronic anticoagulant therapy was used to match patients. A propensity score for each patient was predicted via multivariable logistic regression analysis using all available demographic and comorbidity data. The final model was selected by sequentially minimizing Akaike’s information criterion (AIC) and the Bayesian information criterion (BIC), and variance inflation factor (VIF) > 10 was used to exclude multicollinear covariates. The area under the receiver operating characteristic curve (AUC) and the Hosmer–Lemeshow goodness of fit test were evaluated for this model. Nearest-neighbor matching without replacement using a standard caliper of 0.2 was employed to 1:1 match the cohorts. Appropriate post-match balance was assessed by ensuring that all covariates had a standardized mean difference (SMD) of < 0.10.

### Statistical analysis

All patient demographics, hospital factors, and rates of medical comorbidities were compared between matched groups. The χ^2^ test was used for categorical variables, whereas the Mann–Whitney U or Student’s t-tests were used for continuous variables where appropriate after considering normality and homoscedasticity of the data. To account for residual confounding related to the odds of study outcomes after matching, multivariable logistic regression models on primary and secondary outcomes were conducted, including patient and hospital level data as potential confounders. Model selection was conducted by sequentially minimizing AIC and BIC, while excluding multicollinear covariates. Significance was defined as a Bonferroni corrected *p* < 0.025, and all statistical analyses were conducted using Stata (version 17.0; StataCorp).

### Source of funding

There was no funding source for this study.

## Results

A total of 30,590 patients met inclusion criteria, of which 7665 (25.06%) had a history of chronic anticoagulation. After matching on factors most predictive of having a history of chronic anticoagulation (AUC = 0.827, Hosmer–Lemeshow *p*-value = 0.327; Supplemental Digital Content 1). Prior to matching, the two cohorts were significantly different in the majority of demographic and comorbidity factors, with large differences in the rates of prior history of cardiac arrhythmia and thromboembolic disease (Supplemental Tables 2, 3).

Matching on the propensity score identified 7218 patients on chronic anticoagulation and 7218 patients without a history of chronic anticoagulation with common support. After matching, both groups of chronic anticoagulation use and no history of chronic anticoagulation use demonstrated a mean age of 82 years, with the majority of patients being female (60.86% vs. 60.06% respectively), White (92.3% vs. 92.24% respectively), and unmarried (57.12% vs. 56.26% respectively) (Table 1). All other hospital factors and comorbidities were not significantly different between groups and were well balanced (Table 2 and Supplemental Table 4).

**Table 1:** Demographic factors after matching

	Chronic anticoagulation		No anticoagulation		
	N = 7218		N = 7218		
	Average	SD	Average	SD	SMD
Age (years)	81.59	7.1	82	7.15	0.071
Length of stay (days)	6.37	3.67	6.26	4.15	0.028

	N	%	N	%	
Female sex	4393	60.86%	4335	60.06%	0.016
*Race*					
Asian	78	1.08%	93	1.29%	0.024
Black	228	3.16%	302	4.18%	
Other	230	3.19%	164	2.27%	
Unknown	68	0.94%	49	0.68%	
White	6662	92.30%	6658	92.24%	
*Marital status*					
Married	2724	37.74%	2,812	38.96%	0.022
Other	415	5.75%	389	5.39%	
Single	4123	57.12%	4061	56.26%	
Unknown	4	0.06%	4	0.06%	

SD, standard deviation; SMD, standardized mean difference

**Table 2:** Hospital factors after matching

		Chronic anticoagulation		No anticoagulation		
		N = 7218		N = 7218		
		N	%	N	%	SMD
Medicare insurance	Yes	6,902	95.62%	6,937	96.11%	0.023
Bed size	< 100	438	6.07%	562	7.79%	0.042
	100–199	1065	14.75%	942	13.05%	
	200–299	1406	19.48%	1570	21.75%	
	399–399	1227	17.00%	1203	16.67%	
	400–499	987	13.67%	918	12.72%	
	> 500	2143	29.69%	2071	28.69%	
Urban versus rural	Urban	5753	79.70%	5686	78.78%	0.023
Teaching status	Yes	3564	49.38%	3510	48.63%	0.015
Emergency admission status	Yes	6017	83.36%	6040	83.68%	0.008
Region	Midwest	1830	25.35%	1523	21.10%	0.059
	Northeast	986	13.66%	1240	17.18%	
	South	4264	59.07%	4306	59.66%	
	West	186	2.58%	197	2.73%	

SD, standard deviation; SMD, standardized mean difference

The matched cohort of patients with a history of chronic anticoagulant use had significantly higher rates of non-infectious wound complications (2.15% vs. 1.29%, *p* < 0.001), with higher rates of irrigation and debridement for both non-infectious (1.41% vs. 0.67%, *p* < 0.001) and infectious (0.85% vs. 0.50%, *p* < 0.011) surgical site complications (Table 3). Rates of surgical site infection were not significantly different between groups (1.73% vs. 1.30%, *p* = 0.111). Significantly more patients on anticoagulation also experienced a protracted length of stay at the hospital (49.06% vs. 45.46%, *p* < 0.001). However, fewer aggregate medical complications were observed (34.28% vs. 39.00%, *p* < 0.001).

After adjusting for confounding variables, hip fracture patients on chronic anticoagulation had significantly higher odds of non-infectious (adjusted odds ratio [aOR]: 1.65, 95% confidence interval [95% CI] 1.27–2.15, *p* < 0.001) and infectious (aOR: 1.38, 95% CI 1.05–1.81, *p* < 0.021) wound complications, as well as over two times greater odds of requiring operative irrigation and debridement for non-infectious (aOR: 2.15, 95% CI 1.51–3.04, *p* < 0.001) and infectious (aOR: 2.16, 95% CI 1.52–3.07, *p* < 0.001) wound complications. Chronically anticoagulated patients were also more likely to have a longer hospital course (aOR: 1.19, 95% CI 1.11–1.27, *p* < 0.001), however were significantly less likely to have postoperative medical complications (aOR: 0.83, 95% CI 0.77–0.89, *p* < 0.001). The odds of intraoperative transfusion, thrombotic complications, inpatient mortality, SNF discharge, and readmission were not significantly different between the two groups (all *p* > 0.05) (Table 4).

**Table 3:** Comparison of primary and secondary study outcomes between groups

	Chronic anticoagulation		No anticoagulation		
	N = 7218		N = 7218		
Complication	N	%	N	%	*P* value
Non-infectious wound complication	155	2.15%	93	1.29%	** < 0.001**
Infectious wound complication	125	1.73%	94	1.30%	0.111
Irrigation and debridement for non-infectious wound complication	102	1.41%	48	0.67%	** < 0.001**
Irrigation and debridement for infectious wound complication	61	0.85%	36	0.50%	**0.011**
Intraoperative transfusion	268	3.71%	237	3.28%	0.16
Medical complications	2474	34.28%	2,815	39.00%	** < 0.001**
Thrombotic complications	1115	15.45%	1,057	14.64%	0.177
Inpatient mortality	254	3.52%	268	3.71%	0.533
Protracted length of stay	3541	49.06%	3,281	45.46%	** < 0.001**
Discharge to skilled nursing facility	4770	66.08%	4,782	66.25%	0.834
Readmission	887	12.29%	850	11.78%	0.344

**Table 4:** Multivariable logistic regression of primary and secondary study outcomes

Complication	aOR	95%-confidence interval	*P* value
Non-infectious wound complication	1.65	1.27, 2.15	** < 0.001**
Infectious wound complication	1.38	1.05, 1.81	**0.021**
Irrigation and debridement for non-infectious wound complication	2.15	1.51, 3.04	** < 0.001**
Irrigation and debridement for infectious wound complication	2.16	1.52, 3.07	** < 0.001**
Intraoperative transfusion	1.09	0.91, 1.30	0.361
Medical complications	0.83	0.77, 0.89	** < 0.001**
Thrombotic complications	1.05	0.96, 1.16	0.296
Inpatient mortality	0.95	0.79, 1.13	0.542
Protracted length of stay	1.19	1.11, 1.27	** < 0.001**
Discharge to skilled nursing facility	1.01	0.94, 1.09	0.74
Readmission	1.05	0.95, 1.17	0.316

aOR, adjusted odds ratio; Bold values indicate p<0.025

## Discussion

In this retrospective database study of 7218 older adults on chronic anticoagulant therapy undergoing HA for femoral neck fracture, anticoagulated patients were roughly 50% more likely to have a noninfectious or infectious wound complication. They were subsequently over twice as likely to require debridement for the wound complication and 1.19 times more likely to have a prolonged hospital stay. Notably, the odds of intraoperative transfusion, thrombotic complications, discharge disposition differences, and readmission/ mortality were not different between the two groups after implementing our rigorous matching algorithm.

Wound complication following joint replacement surgeries is a devastating outcome that can lead to longer postoperative morbidity, lower patient reported outcome scores, revision surgeries, and higher overall healthcare costs [14]. The association between wound complications and anticoagulation has been well documented in pelvic and hip surgeries. McDougal et al. retrospectively reviewed 268 patients who underwent total hip arthroplasty and determined an increase in deep infection, wound hematoma, and superficial infection in the perioperatively anticoagulated patients [15]. Similarly, Patel et al. noted a higher rate of wound infection and drainage in total hip arthroplasty for patients treated with low molecular weight heparin [16]. Hematomas and persistently draining wounds have been associated with wound infection previously, both likely contributing to the chronic anticoagulation-associated surgical site infectious sequelae [14, 16, 17]. In line with these investigations, our study controlled for comorbidities and similarly demonstrated that anticoagulated patients were more likely to have both infectious and noninfectious wound complications that then required concomitant debridement.

After matching on the propensity for having a history of chronic anticoagulation, we found a higher rate of medical complications in patients not treated with anticoagulation. As demonstrated in Gleason et al., hip fracture patients on chronic anticoagulation therapy generally have a higher Charleston comorbidity index [18]. Despite these differences, medical complications after hip surgery between the two groups are generally comparable and insignificant [18, 19]. Our study compared those with anticoagulation therapy to those with similar indications for therapy and not treated with anticoagulants. The most common comorbidities were cardiac arrythmia and complicated hypertension at 71% and 48% prevalence respectively, both of which may be an indicator for anticoagulation treatment [20]. These patients who receive chronic anticoagulants may have better routine medical care and monitoring to ensure stable health as opposed to those with similar comorbidities and indications for anticoagulation treatment. Thus, the anticoagulants may serve as a surrogate for medical care, and these patients may have a better post-operative clinical course.

Preoperative management of anticoagulants requires determining risks associated with discontinuing anticoagulants versus surgical delay. Current guidelines recommend a time to surgery within 48 h for patients presenting with hip fractures, but anticoagulants are often a source of delays [21, 22]. Data regarding chronic use of anticoagulant effects on post-operative mortality is heterogeneous and intertwined with the consequential delay to surgery [22]. Numerous studies have identified higher rates of surgical delay in older adult hip fracture patients on anticoagulation therapy and a consequential higher rate of postoperative mortality [23, 24]. In contrast, Saliba et al. found direct oral anticoagulants (DOACs) reduced mortality in hip fracture patients despite increasing surgical delay [25]. Gleason et al. and Ueoka et al. found no significant change in 1 year mortality in hip fracture patients [18, 19]. Our study uniquely compares patients matched for comorbidities and found no impact on 90-day mortality from anticoagulant use despite a protracted length of stay. This adds to the growing literature on the impact of anticoagulants and supports non-delayed surgery for patients on chronic anticoagulation therapy.

Analogous to the lack of impact on mortality, anticoagulants also did not affect frequency of readmissions or discharge to a skilled nursing facility. Within these patients matched for comorbidities, the use of anticoagulants was not relevant to clinical condition within the hospital to dictate disposition status nor clinical course after discharge to warrant readmission. These findings are consistent with Gleason et al. and Lotti et al. who also demonstrated an insignificant effect on readmission rates [18, 26]. However, they differ from Franklin et al. and Levack et al., which indicated a higher 30-day readmission with DOACs and 90-day readmission with warfarin, respectively, in hip fracture patients [27, 28]. Studies that utilized an unmatched patient cohort unsurprisingly found a higher amount of post-surgical acuity care required for the anticoagulated patients, including ICU stay and discharge to a skilled nursing facility, despite similar surgical outcomes and complications [29]. A supporting finding by Korotkov et al. found more cardiovascular disease in anticoagulated patients but comparable surgical outcomes, perioperative complications, and functional independence [30]. Ultimately, these articles propose that discharge status and readmission rates are most impacted by comorbidities, which are associated with anticoagulant use.

Decisions to continue anticoagulants requires weighing the risk of bleeding to thrombotic complications. Our study found no significant impact on intraoperative transfusion or thrombotic complications. The landmark BRIDGE randomized control trial evaluated patients admitted for various nonemergent surgical procedures, including orthopedic procedures. This investigation determined that risks of bleeding outweighed risks of thrombotic complications for patients bridged with heparin during perioperative warfarin discontinuation [31]. More specific investigations related to orthopedic procedures have found similar results. In hip fracture patients treated with HA, Xu et al. similarly noted a larger amount of surgical blood loss but also described a consequential higher rate of transfusions. Frequency of postoperative thromboembolism was not significantly impacted [32]. Levack et al. compared low energy hip fracture patients on DOACs and reported no difference in transfusion rates or thrombotic complications [33]. A systematic analysis by Cheung reported mixed findings in the literature with the majority indicating no significant difference in thromboembolism, stroke, or transfusions [34]. Likely the heterogeneity in transfusion rates derives from controversial guidelines and thresholds for transfusions [35]. Our study adds to the heterogeneous literature on thrombotic and bleeding risks of anticoagulant use, delineating the associated risks of non-delayed operative treatment in patients admitted with chronic anticoagulant use.

This study was limited by its retrospective review of a large database with potential for misclassification bias and incomplete data. Importantly, the PHD represents an all-payer database comprising approximately one quarter of annual United States inpatient admissions from more than 1000 hospitals. Overall, < 1% of patient records have missing information such as demographics, and diagnostic information are missing in approximately 0.01% of files per the PHD data documentation. Another key consideration is that given our approach in identifying patients on chronic anticoagulation via ICD-10-CM coding, we were limited in categorizing the specific types of anticoagulants and determining the exact duration of therapy. Furthermore, it remains unknown whether these patients had their anticoagulants discontinued prior to surgery, the duration of any such discontinuation, or when these agents were resumed postoperatively. While this may introduce confounding, the large sample size, the propensity matching for comorbidities, and the consistency with surgical procedure reconciliate variability in this population. Moreover, this uncertainty reflects clinical practice such that clinical guidelines regarding the type and duration of anticoagulation use, as well as the associated risk of postoperative complications, are not well delineated. Further investigations would require a randomized trial or prospective study to determine the impact of anticoagulants more precisely.

## Conclusion

The present data provide compelling evidence that chronic anticoagulation confers an independent risk of wound complications in patients undergoing HA for hip fracture in older adults. In line with the heterogeneous literature, our study found no significant impact by anticoagulants on mortality, readmissions, thrombotic risk, or bleeding risk. This study advocates for non-delayed early operative treatment for hip fracture patients with extra precaution towards surgical site issues. The small increased risk of wound complications is likely compensated by early mobilization and avoiding complications of bed rest.

## Supplementary Information

Below is the link to the electronic supplementary material.

Supplementary Material 1

## Data Availability

Data is provided by the PREMIER health database.
