# Supplementary material for: Chronic anticoagulation therapy associated with increased complications following hemiarthroplasty in hip fracture patients
Source: Eur J Orthop Surg Traumatol. 2025 Nov 4;35(1):450. doi: 10.1007/s00590-025-04578-w (PMC12586207; doi:10.1007/s00590-025-04578-w)
Supplement: Supplementary file 1 — Supplementary Material 1 [file 590_2025_4578_MOESM1_ESM.docx]

Supplementary Table 1.

| **Diagnosis** | **ICD-10-CM code** |
| --- | --- |
| Seroma, hematoma, and wound dehiscence | M96.842, T81.3, L76.22, M96.84 |
| Superficial and deep surgical site infection | T81.41, T81.42, T81.43, T82.42, T82.43 |
| Acute kidney injury, acute respiratory failure, atrial fibrillation with rapid ventricular response, urinary tract infection, pneumonia, and sepsis | J12, J13, J14, J15, J16, J18, J60, J95.851, J96, J80, J81.0, N17.0, N17.1, N17.2, N17.8, N17.9, N30.00, N30.01, N30.80, N30.81, N30.90, N30.91, N39.0, A41.01, A41.02, A41.2, A41.3, A41.4, A41.50, A41.51, A41.52, A41.53, A41.59, A41.81, A41.89, A41.9, R65.20, I48.0, J96, J80, J81.0 |
| Myocardial infarction, cerebrovascular accident, and venous thromboembolism | I82.4, I26.02, I26.09, I26.92, I26.93, I26.94, I26.99, I61, I63, I97.811, I97.821, I21.01, I21.02, I21.09, I21.11, I21.19, I21.21, I21.29, I21.3, I21.4, I21.9, I21.A1, I21.A9 |
| **Procedure** | **CPT code** |
| Irrigation and debridement | 26990, 11044, 11043 11042, 11045, 11046, 11047 |
| Transfusion | 36430 |

Supplementary Table 2.

|  | | **Chronic Anticoagulation**  **N = 7,665** | | **No Anticoagulation**  **N = 22,925** | |  |
| --- | --- | --- | --- | --- | --- | --- |
|  |  | **Average** | **Standard Deviation (SD)** | **Average** | **SD** | **P-value** |
| **Age (years)** | | 80.92 | 7.38 | 79.54 | 8.32 | **<0.001** |
| **Length of Stay (days)** | | 6.34 | 3.62 | 5.70 | 3.61 | **<0.001** |
|  | | **N** | **%** | **N** | **%** |  |
|  | |  |  |  |  |  |
| **Female Sex** | | 4,552 | 59.39% | 15,942 | 69.54% | **<0.001** |
| **Race** | Asian | 78 | 1.02% | 438 | 1.91% | **<0.001** |
|  | Black | 245 | 3.20% | 1,148 | 5.01% |  |
|  | Other | 240 | 3.13% | 924 | 4.03% |  |
|  | Unknown | 69 | 0.90% | 271 | 1.18% |  |
|  | White | 7,033 | 91.75% | 20,144 | 87.87% |  |
| **Marital Status** | Married | 2,890 | 37.70% | 8,386 | 36.58% | **0.132** |
|  | Other | 445 | 5.81% | 1,470 | 6.41% |  |
|  | Single | 4,326 | 56.44% | 13,057 | 56.96% |  |
|  | Unknown | 4 | 0.05% | 12 | 0.05% |  |
| **Medicare Insurance** | Yes | 7,295 | 95.17% | 21,309 | 92.95% | **<0.001** |
| **Bed size** | <100 | 449 | 5.86% | 1,678 | 7.32% | **<0.001** |
|  | 100-199 | 1,112 | 14.51% | 3,032 | 13.23% |  |
|  | 200-299 | 1,515 | 19.77% | 4,650 | 20.28% |  |
|  | 399-399 | 1,288 | 16.80% | 3,878 | 16.92% |  |
|  | 400-499 | 1,080 | 14.09% | 2,423 | 10.57% |  |
|  | >500 | 2,221 | 28.98% | 7,264 | 31.69% |  |
| **Urban vs. Rural** | Urban | 6,043 | 78.84% | 17,835 | 77.80% | **0.056** |
| **Teaching Status** | Yes | 3,761 | 49.07% | 10,801 | 47.11% | **0.003** |
| **Emergency Admission Status** | Yes | 6,315 | 82.39% | 19,793 | 86.34% | **<0.001** |
| **Region** | Midwest | 1,914 | 24.97% | 4,888 | 21.32% | **<0.001** |
|  | Northeast | 1,023 | 13.35% | 3,695 | 16.12% |  |
|  | South | 4,537 | 59.19% | 13,471 | 58.76% |  |
|  | West | 191 | 2.49% | 871 | 3.80% |  |

Supplementary Table 3.

|  | **Chronic Anticoagulation**  **N = 7,665** | | **No Anticoagulation**  **N = 22,925** | | **P-value** |
| --- | --- | --- | --- | --- | --- |
|  | **N** | **%** | **N** | **%** |  |
| Congestive Heart Failure | 2,966 | 38.70% | 4,046 | 17.65% | **<0.001** |
| Cardiac Arrhythmia | 5,432 | 70.87% | 5,797 | 25.29% | **<0.001** |
| Myocardial Infarction | 1,172 | 15.29% | 2,118 | 9.24% | **<0.001** |
| Valvular Disease | 538 | 7.02% | 311 | 1.36% | **<0.001** |
| Pulmonary Hypertension | 945 | 12.33% | 1,241 | 5.41% | **<0.001** |
| Chronic Pulmonary Disease | 2,251 | 29.37% | 5,372 | 23.43% | **<0.001** |
| Peripheral Vascular Disease | 1,347 | 17.57% | 1,948 | 8.50% | **<0.001** |
| Hypertension | 3,209 | 41.87% | 11,280 | 49.20% | **<0.001** |
| Complicated Hypertension | 3,750 | 48.92% | 7,003 | 30.55% | **<0.001** |
| Cerebrovascular Accident | 2,383 | 31.09% | 3,328 | 14.52% | **<0.001** |
| Hemiplegia/Paraplegia | 27 | 0.35% | 142 | 0.62% | **0.006** |
| Other neurological disorders | 1,235 | 16.11% | 3,659 | 15.96% | 0.754 |
| Diabetes, uncomplicated | 797 | 10.40% | 2,436 | 10.63% | 0.574 |
| Diabetes, complicated | 1,573 | 20.52% | 3,492 | 15.23% | **<0.001** |
| Hypothyroidism | 1,998 | 26.07% | 5,481 | 23.91% | **<0.001** |
| Renal Failure | 2,484 | 32.41% | 5,597 | 24.41% | **<0.001** |
| Liver Disease | 187 | 2.44% | 750 | 3.27% | **<0.001** |
| Chronic Peptic Ulcer Disease | 24 | 0.31% | 104 | 0.45% | 0.099 |
| Blood Loss Anemia | 131 | 1.71% | 436 | 1.90% | 0.279 |
| Deficiency Anemia | 578 | 7.54% | 1,385 | 6.04% | **<0.001** |
| Coagulopathy | 918 | 11.98% | 2,035 | 8.88% | **<0.001** |
| Venous Thromboembolism | 1,174 | 15.32% | 975 | 4.25% | **<0.001** |
| Fluid and Electrolyte Disorders | 2,923 | 38.13% | 8,664 | 37.79% | 0.594 |
| Rheumatic Disease | 317 | 4.14% | 1,045 | 4.56% | 0.120 |
| HIV/AIDS | 2 | 0.03% | 23 | 0.10% | **0.049** |
| Lymphoma | 119 | 1.55% | 238 | 1.04% | **<0.001** |
| Solid Tumor | 298 | 3.89% | 1,042 | 4.55% | **0.015** |
| Metastatic Cancer | 171 | 2.23% | 663 | 2.89% | **0.002** |
| Obesity | 695 | 9.07% | 1,335 | 5.82% | **<0.001** |
| Weight Loss | 639 | 8.34% | 2,267 | 9.89% | **<0.001** |
| Alcohol Abuse | 159 | 2.07% | 865 | 3.77% | **<0.001** |
| Drug Abuse | 57 | 0.74% | 344 | 1.50% | **<0.001** |
| Psychoses | 59 | 0.77% | 351 | 1.53% | **<0.001** |
| Depression | 1,452 | 18.94% | 4,240 | 18.50% | 0.383 |
